# Purpuric Plaques—Dermoscopic and Histopathological Correlation of Cutaneous Angiosarcoma

**DOI:** 10.5826/dpc.1004a84

**Published:** 2020-10-26

**Authors:** Daniel W. Cole, Tomas Huerta, Aleodor Andea, Trilokraj Tejasvi

**Affiliations:** 1 Wayne State University School of Medicine, Detroit, MI, USA; 2 Department of Dermatology, University of Michigan, Ann Arbor, MI, USA; 3 Department of Pathology, University of Michigan, Ann Arbor, MI, USA

**Keywords:** cutaneous angiosarcoma, dermoscopy, nonmelanocytic skin cancer, histopathology

## Introduction

Cutaneous angiosarcoma (cAS) is an aggressive mesenchymal neoplasm of vascular endothelial cells that typically presents in middle-aged to elderly individuals as an expanding ecchymotic patch on the head or neck region [1]. As more dermoscopic features of uncommon skin tumors are described in the literature, the importance of establishing specific criteria for the diagnosis of high-risk lesions is paramount. In this article, we delineate the dermoscopic features of cAS and their correlation to its histopathologic findings.

## Case Presentation

**Figure 1:**
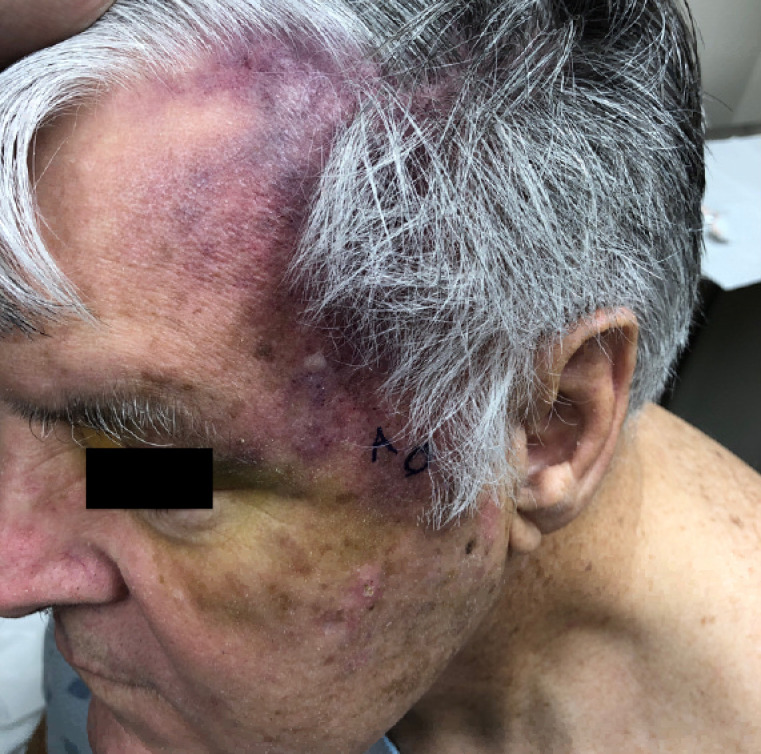
Exam showed a red-purple and yellow-black patch on the left periorbital skin that expanded to involve the left forehead, frontal, temporal, and parietal scalp.

**Figure 2:**
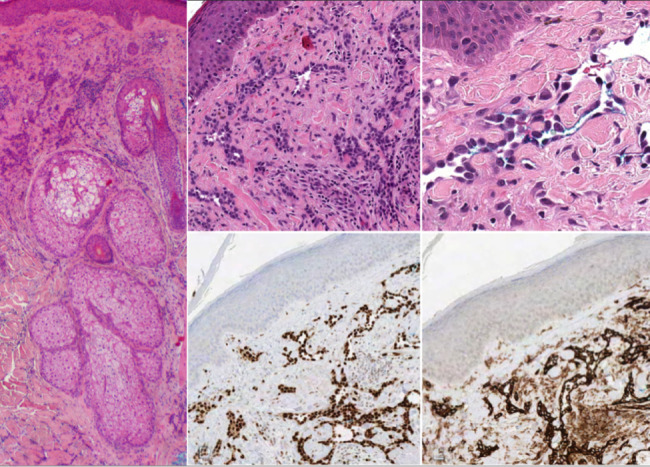
Punch biopsy. (A) Lower power view of tumor architecture sparing pilosebaceous units. (B) Infiltrative vascular neoplasm involving the dermis and extending to the peripheral margin. (C) Irregular interanastomosing vascular channels lined by atypical endothelial cells. (D) Immunohistochemical stain for ERG highlights the atypical endothelial cells. (E) Immunohistochemical stain for CD31 highlights the atypical endothelial cells.

Our patient is a 74-year-old man who presented to the clinic with a 2-month history of a red-purple and yellow-black patch on his left periorbital skin expanding to involve his left forehead, frontal, temporal, and parietal scalp (Figure 1). Palpable edema was appreciated periorbitally. A punch biopsy was performed and demonstrated a high-grade infiltrative vascular neoplasm involving the dermis consistent with a diagnosis of angiosarcoma (Figure 2). CD31 and ERG immunohistochemical stains highlighted the atypical endothelial cells confirming the diagnosis.

**Figure 3:**
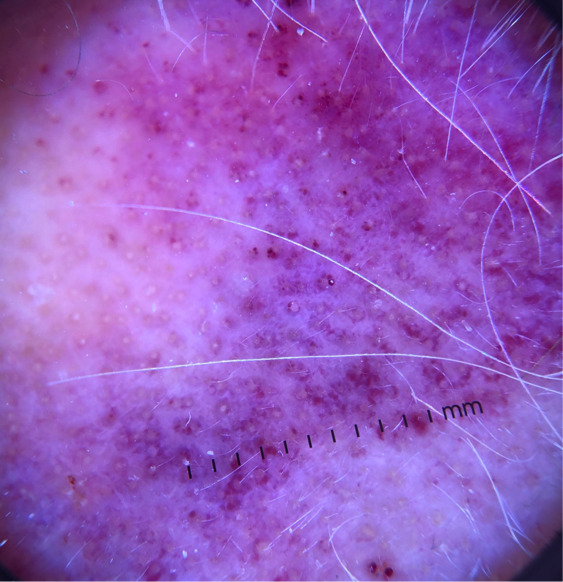
Dermoscopy demonstrating dark red and purple structureless zones on a light red background with a violaceous hue seen in more densely covered areas. Dark red and purple clods are clustered around follicular openings that appear as yellow and white circles. Thick, polygonal and perpendicular white lines are scattered throughout.

The dermoscopy image from our patient demonstrated dark red and purple structureless zones on a light red background. A violaceous hue was also observed in more densely covered areas (Figure 3). There were thick white, perpendicular, and polygonal lines scattered throughout. Centrally we noted many dark red and purple dots and clods clustered around follicular openings, which appeared as white and yellow circles. This observation is a unique pattern in cAS. Interpreted in conjunction with the histopathologic images (Figure 2), the perifollicular clods represent vascular channels that remarkably spare pilosebaceous units despite the infiltrative, aggressive nature of these tumors. The varying color gradations correlate with the position of the vascular structures in the dermis, with superficial dermal vascular structures appearing red to dark red, and deeper dermal involvement (nearly to the level of the subcutis) imparting a violaceous hue to the lesion.

## Conclusions

In previous reports, cAS is dermoscopically characterized by structureless, patchy pink, red, and purple-blue areas with yellowish round clods corresponding to follicular openings [2]. These color gradations represent various tumor components: pink areas are highly cellular, red polymorphic areas consist of telangiectasias or vascular channels, and dark red to purple areas may be organizing thrombus, hemorrhage, or dilated vascular structures [1]. Additionally, white lines may be present within these areas or at the periphery of nodular foci and are histopathologically correlated to fibrous septa between neoplastic vascular spaces [2]. The perifollicular clods observed in our patient represent tumor vascular channels.

Although rare, the aggressive nature of this malignancy underscores the importance of early diagnosis. The presence of many dark red and purple clods surrounding follicular openings, along with previously described features and a suggestive history, should prompt high clinical suspicion of cAS and histopathologic evaluation.
